# No Efficacy of the Combination of Lopinavir/Ritonavir Plus Hydroxychloroquine Versus Standard of Care in Patients Hospitalized With COVID-19: A Non-Randomized Comparison

**DOI:** 10.3389/fphar.2021.621676

**Published:** 2021-04-22

**Authors:** Roberta Gagliardini, Alessandro Cozzi-Lepri, Andrea Mariano, Fabrizio Taglietti, Alessandra Vergori, Amina Abdeddaim, Francesco Di Gennaro, Valentina Mazzotta, Alessandra Amendola, Giampiero D’Offizi, Fabrizio Palmieri, Luisa Marchioni, Pierluca Piselli, Chiara Agrati, Emanuele Nicastri, Maria Rosaria Capobianchi, Nicola Petrosillo, Giuseppe Ippolito, Francesco Vaia, Enrico Girardi, Andrea Antinori

**Affiliations:** ^1^ National Institute for Infectious Diseases Lazzaro Spallanzani IRCCS, Rome, Italy; ^2^ Centre for Clinical Research, Epidemiology, Modelling and Evaluation (CREME), Institute for Global Health, University College London, London, United Kingdom

**Keywords:** SARS-CoV-2, antivirals, drug repurposing, viral shedding, invasive ventilation

## Abstract

**Objectives:** No specific treatment has been approved for COVID-19. Lopinavir/ritonavir (LPV/r) and hydroxychloroquine (HCQ) have been used with poor results, and a trial showed advantages of combined antiviral therapy vs. single antivirals. The aim of the study was to assess the effectiveness of the combination of antivirals (LPV/r and HCQ) or their single use in COVID-19 hospitalized patients vs. standard of care (SoC).

**Methods:** Patients ≥18 years with SARS-CoV-2 infection, defined as positive RT-PCR from nasal/oropharyngeal (NP/OP) swab or positive serology, admitted at L. Spallanzani Institute (Italy) were included.

**Primary endpoint:** time to invasive ventilation/death. Secondary endpoint: time to two consecutive negative SARS-CoV-2 PCRs in NP/OP swabs. In order to control for measured confounders, a marginal Cox regression model with inverse probability weights was used.

**Results:** A total of 590 patients were included in the analysis: 36.3% female, 64 years (IQR 51–76), and 91% with pneumonia. Cumulative probability of invasive ventilation/death at 14 days was 21.2% (95% CI 17.6, 24.7), without difference between SOC, LPV/r, hydroxychloroquine, HCQ + LPV/r, and SoC. The risk of invasive ventilation/death in the groups appeared to vary by baseline ratio of arterial oxygen partial pressure to fractional inspired oxygen (PaO2/FiO2). Overall cumulative probability of confirmed negative nasopharyngeal swabs at 14 days was 44.4% (95% CI 38.9, 49.9), without difference between groups.

**Conclusion:** In this retrospective analysis, we found no difference in the rate of invasive ventilation/death or viral shedding by different strategies, as in randomized trials performed to date. Moreover, even the combination HCQ + LPV/r did not show advantages vs. SoC.

## Introduction

In December 2019, an outbreak of viral pneumonia cases of unknown cause was identified in Wuhan, China. A novel coronavirus was quickly identified in some of these patients and it has been designated as severe acute respiratory syndrome coronavirus 2 (SARS-CoV-2) (European Centre for Disease Prevention and Control, 2020). Currently, there are no approved therapeutic agents available for SARS-CoV-2, and great efforts have been unfolded for the discovery of possible treatment strategies. Many repurposed drugs have shown some preclinical activity against SARS-CoV-2 and have been experimented *in vivo* (Tobaiqy et al., 2020). Prompt identification and implementation of life support therapies are pivotal steps in order to prevent the spreading of the infection and improve patient’s clinical outcome. Some data about treatment from observational studies, compassionate use programs, and few RCT results are available up-to-date.

Among the antiviral strategies, the antiretroviral drug lopinavir/ritonavir (LPV/r) had already demonstrated activity against SARS-CoV-2 and Middle East respiratory syndrome (MERS)-CoV (Ford et al., 2020). Two initial RCTs about lopinavir/ritonavir for treatment of SARS-Cov-2 showed inconclusive results. A small randomized, controlled, open-label trial conducted in China did not observe any benefit of lopinavir/ritonavir treatment vs. standard care in reducing the time to clinical improvement in hospitalized adult patients with severe COVID-19. However, the study appeared to be underpowered and post hoc analyses showed accelerated clinical recovery (16.0 vs. 17.0 days) and reduced mortality (19.0 vs. 27.1%) in the subgroup of patients treated within 12 days after the onset of symptoms (Cao et al., 2020). Another very small RCT comparing lopinavir/ritonavir vs. arbidol for treating patients with mild/moderate COVID-19 showed no differences in term of viral clearance, symptoms resolution, and radiological improvement between the arms (Li et al., 2020).

Follow-up retrospective studies did not show evidence of effectiveness of lopinavir/ritonavir and of other antiretrovirals, as recently systematically reviewed (Ford et al., 2020).

A possible antiviral activity against SARS-CoV-2 of hydroxychloroquine (HCQ) has been supposed. This drug inhibits the glycosylation of ACE II, the receptor used by SARS-CoV-2 to enter the cells, and could result in a reduced ligand recognition and internalization of the virus (Vincent et al., 2005). This activity, together with the best known immunomodulatory and anti-inflammatory effects, yielded HCQ an interesting drug in this contest, but the most recent results showed lack of efficacy.

A review of seven clinical trials has shown contrasting results, but the analyzed studies posed significant risk for bias in the randomization process, in measurement of outcomes, or in deviations from planned interventions (Chowdhury et al., 2020). Recent data from the RECOVERY trial, a large randomized study, showed no evidence of benefit for mortality or other outcomes (duration of hospitalization and need for invasive ventilation) of HCQ treatment in hospitalized patients with COVID-19. Indeed, day-28 mortality was reported as 25.7% with HCQ and 23.5% with comparator (hazard ratio 1.11, 95% CI 0.98–1.26, *p* = 0.10), so the investigators announced closure of the HCQ arm due to lack of effectiveness (Recovery Randomised Evaluation of COVID-19 Therapy, 2020a). Similarly, also the LPV/r arm in RECOVERY was halted for the same reasons (World Health Organization, 2020).

Even the ORCHID study, a clinical trial evaluating the safety and effectiveness of hydroxychloroquine for the treatment of hospitalized adults with COVID-19, has been halted by NIH (NIH, 2020b). Solidarity trial showed no effect of hydroxychloroquine or lopinavir/ritonavir on hospitalized patients with COVID-19, as indicated by overall mortality, initiation of ventilation, and duration of hospital stay (WHO Solidarity Trial Consortium et al., 2020). Even in terms of antivirals’ effectiveness on ending SARS-COV-2 shedding, conflicting results about the clinical role of HCQ have been published. In fact, one report observed a positive impact on viral shedding (Huang et al., 2020), but a large RCT showed no difference in probability of negative conversion (Tang et al., 2020). Currently, NIH guidelines recommend against the use of HCQ or LPV/r for treatment of COVID-19 because of lack of effectiveness (NIH, 2020a).

However, triple combination of interferon beta-1b, lopinavir/ritonavir, and ribavirin resulted to be superior to lopinavir/ritonavir alone in alleviating symptoms and shortening the duration of viral shedding and hospital stay in patients with mild-to-moderate COVID-19 (Hung et al., 2020). Thus, these data provided a proof of concept for the possible synergic effect of using a combination of two or more antivirals to improve effectiveness like that seen for other infections such as HIV. More recently, an in silico approach proposed a possible synergistic effect of 16 compounds with independent mechanism of action in SARS-CoV-2 (Bobrowski et al., 2021).

The purpose of this study was to explore the difference in effectiveness of single antivirals (lopinavir/ritonavir and HCQ) and their combination when compared to current standard of care (SoC) in COVID-19 hospitalized patients, by emulating a RCT using the observational retrospective data of the INMI COVID-19 database.

## Materials and Methods

We conducted a retrospective cohort study on the INMI COVID-19 database of L. Spallanzani Institute in Rome (Italy) that contains data from consecutive hospitalized patients (≥18 years of age) who had a positive test result for the SARS-CoV-2 virus at any time during their hospitalization from January 29 to June 13, 2020. Participants’ follow-up of those not yet discharged was administratively censored on July 1st^,^ 2020. INMI COVID-19 database was approved by the local INMI, Rome Ethical Committee and patients provided written informed consent. The study was performed in accordance with the Declaration of Helsinki. INMI COVID-19 database retrieves epidemiological, demographic, clinical, and laboratory data of patients, as well as therapy prescribed (antiviral, immunomodulatory drugs, oxygen therapy, and need for ventilation) for COVID-19 patients.

Patients were included in this study if the following inclusion criteria were satisfied: ≥18 years of age, a diagnosis of SARS-CoV-2 infection, defined as positive RT-PCR from nasal/oropharyngeal (NP/OP) swab or positive serology, and admitted at INMI L. Spallanzani Institute.

This is a retrospective study which was conducted in exceptional conditions during the first wave of COVID-19 pandemic in Italy, so sample size was not preplanned.

In all included patients, diagnosis of SARS-CoV-2 infection was confirmed by the detection of SARS-CoV-2 RNA through real-time polymerase chain reaction (RT-PCR) targeting the E and RdRp viral genes on NP/OP swab. Subsequently, during the hospitalization, all patients underwent follow-up NP/OP swab to assess the clearance of viral RNA. The timing of follow-up NP/OP swab was variable, according to treating physician’s judgment.

We compared four treatment strategies initiated after hospital admission: 1) starting hydroxychloroquine; 2) starting lopinavir/ritonavir; 3) starting hydroxychloroquine plus lopinavir/ritonavir; and 4) a control group receiving none of the previous drugs (standard of care). Standard of care included any supportive therapy: fluids, antibiotics, oxygen supplementation, and any concomitant therapy except for HCQ and LPV/r. Concomitant use of therapy with immunomodulants (e.g., anti-IL6 and anti-JAK), corticosteroids, heparin, and antibiotics (including azithromycin) was controlled for in the analysis. The decision of whether to treat patients with off-label hydroxychloroquine or lopinavir/ritonavir or other drugs was based on local medical consensus, guidelines, and the clinicians’ own opinion.

The most commonly prescribed dosage of HCQ was 400 mg orally bid in the first day, followed by 200 mg bid for a total of 10 days and of lopinavir/ritonavir was 400/100 mg orally bid for 14 days.

The start of follow-up (baseline) for each patient was the first start of any therapy. All patients were followed up from baseline until death, discharge, last available visit, or the administrative censored date of July 1st^,^ 2020, whichever occurred first.

The ratio of arterial oxygen partial pressure (PaO2) to fractional inspired oxygen (FiO2) between ≤300 mmHg was used as marker of severe respiratory disease, according to NIH guidelines (NIH, 2020a), for the stratified analysis.

The primary endpoint of this study was the evaluation of time from starting of therapy to invasive ventilation or death (whichever occurred first).

The secondary endpoints were 1) the evaluation of time from treatment initiation to two consecutive negative SARS-CoV-2 PCRs in nasal/oropharyngeal swabs, without an in-hospital relapse; 2) the evaluation of time from starting of therapy to noninvasive or invasive ventilation or death (whichever occurred first). Noninvasive ventilation includes CPAP or NIV.

For the secondary endpoint of evaluation of viral shedding, patients with diagnosis of COVID-19 infection made by SARS-CoV-2 serology were excluded from the analysis.

For the statistical analysis, chi-square or nonparametric Kruskal–Wallis tests were used to compare categorical or continuous variables in descriptive analysis, respectively. Besides age and PaO2/FiO2, which showed approximately symmetric distribution, all other continuous variables showed skewed distributions and are therefore expressed as median values with interquartile ranges (IQR). Comparison of age and PaO2/FiO2 by the parametric ANOVA test led to identical conclusions (data not shown).

Unweighted Kaplan–Meier curves were used to compare cumulative probabilities of invasive ventilation/death and of confirmed negative NP/OP swabs by treatment group. Stratification for baseline PaO2/FiO2 (0–300 mmHg vs. > 300 mmHg) and interaction test were used to test whether response to treatment groups might differ in subsets of participants.

A marginal Cox regression model with inverse probability of treatment weighting approach (IPW) was used to balance the differences in baseline and time-varying variables between treatment groups. Propensity scores to construct the weights were based on a vector of potential confounders identified a priori on the basis of axiomatic knowledge and previously published results. These included time-fixed variables measured at entry (i.e., gender, age, extent of comorbidity, and duration of symptoms), as well as time-varying confounders affected by initial treatment choice such as intensification by use of azithromycin, anticoagulants, steroids, and immunomodulatory drugs. A double-robust estimator was used, controlling also for potential informative censoring. The assumption of no positivity was checked by inspecting the distribution of the standardized combined weights. In a subset of participants with available data, we further controlled for baseline levels of inflammation and coagulation (CRP, ferritin, and d-dimer). In an additional sensitivity analysis, severity of disease at baseline was controlled using a diagnosis of pneumonia at entry in the study instead of the PaO2/FiO2 level.

An intention-to-treat approach was used. For endpoints including individual components of the composite endpoint (e.g., separately only invasive ventilation or death), the first event occurred was counted.

All statistical analyses were performed with SAS statistical package version 9.4 (Carey NC, United States).

## Results

### Population Characteristics

A total of 590 patients with diagnosis of COVID-19 were included in this analysis (demographic characteristics, signs, and symptoms are shown in Table 1 and biomarkers in Table 2): 36.3% female, median age of 64 years (IQR 51–76), 91% with a diagnosis of pneumonia, median baseline PaO2/FiO2 of 324 (IQR 244–398) mmHg, days from onset of symptoms to hospitalization were 9 (IQR 5–12), and 17.8% with 2 or more comorbidities. The most represented comorbidities were hypertension (44.7%), followed by cardiovascular disease (30%) and cancer (29.9%). The median time from hospital admission to baseline was 0 days (IQR 0–1).

**TABLE 1:** Demographics, comorbidities, sign, and symptoms of the overall population and of the 4 groups.

Characteristic	Intervention					
	LPV/r	HCQ	LPV/r + HCQ	SoC	p-value^a^	Total
	*N* = 124	*N* = 109	*N* = 244	*N* = 113		*N* = 590
Age, years, median (IQR)	64 (53, 75)	69 (55, 79)	61 (50, 74)	69 (49, 82)	0.076	64 (51, 76)
Female gender, *n* (%)	41 (33.1%)	51 (46.8%)	73 (29.9%)	49 (43.4%)	0.006	214 (36.3%)
Baseline Po2/FiO2, median (IQR)	252 (170, 326)	348 (277, 429)	333 (256, 386)	381 (300, 467)	<0.001	324 (244, 398)
Baseline Po2/FiO2 < 300, *n* (%)	53 (66.3%)	28 (35.4%)	64 (37.2%)	17 (26.2%)	<0.001	162 (40.9%)
Pneumonia, *n* (%)	118 (95.2%)	104 (95.4%)	238 (97.5%)	77 (68.1%)	<0.001	537 (91.0%)
Follow-up, days	10 (5, 31)	12 (5, 26)	12 (6, 23)	10 (4, 21)	0.560	11 (5, 23)
≥2 comorbidities^b^	26 (21.0%)	20 (18.3%)	35 (14.3%)	24 (21.2%)	0.287	105 (17.8%)
Comorbidities, *n* (%)						
Diabetes	19 (15.3%)	33 (30.3%)	34 (13.9%)	19 (16.8%)	0.091	105 (17.8%)
Hypertension	48 (38.7%)	68 (62.4%)	109 (44.7%)	39 (34.5%)	0.195	264 (44.7%)
Cardiovascular disease	34 (27.4%)	39 (35.8%)	65 (26.6%)	39 (34.5%)	0.008	177 (30.0%)
Chronic renal insufficiency	8 (6.5%)	8 (7.3%)	8 (3.3%)	9 (8.0%)	0.215	33 (5.6%)
Cancer	10 (14.7%)	43 (53.8%)	37 (23.7%)	26 (31.0%)	0.251	116 (29.9%)
HIV	2 (1.6%)	4 (3.7%)	0 (0.0%)	4 (3.5%)	0.213	10 (1.7%)
Days from symptoms onset to hospitalization, median (IQR)	8 (5, 11)	8 (3, 11)	9 (7, 12)	10 (3, 22)	0.020	9 (5, 12)
Sign and symptoms, *n* (%)						
Fever	101 (82%)	64 (62%)	211 (86%)	65 (62%)	<0.001	441 (77%)
Cough	88 (71.5%)	46 (44.7%)	153 (62.7%)	50 (50.0%)	<0.001	337 (59.1%)
Myalgia	10 (8.1%)	9 (8.7%)	28 (11.5%)	16 (16.0%)	0.338	63 (11.1%)
Conjunctivitis	3 (2.4%)	4 (3.9%)	6 (2.5%)	11 (11.0%)	0.479	24 (4.2%)
Headache	13 (10.6%)	10 (9.7%)	23 (9.4%)	17 (16.7%)	0.535	63 (11.0%)
Dyspnea	53 (43.1%)	36 (35.0%)	72 (29.5%)	44 (42.7%)	0.080	205 (35.8%)
Diarrhea	12 (9.8%)	12 (11.7%)	26 (10.7%)	17 (16.8%)	0.482	67 (11.7%)

^a^ Chi-square or Kruskal–Wallis test as appropriate.

^b^ Asthma, cardiovascular disease, diabetes, hepatic disease, HIV, renal disease, hypertension, cancer, and TB.

**TABLE 2:** Biomarkers and other baseline characteristics.

	Intervention					
	LPV/r	HCQ	LPV/r + HCQ	SoC	p-value^a^	Total
	*N* = 124	*N* = 109	*N* = 243	*N* = 112		*N* = 588
Neutrophils, N	4.2 (2.9, 6.4)	4.0 (2.8, 5.8)	3.7 (2.7, 5.6)	4.8 (3.2, 6.9)	0.031	4.1 (2.8, 5.9)
Neutrophils, %	72.0 (62.3, 82.3)	67.9 (57.9, 77.5)	70.1 (60.3, 79.7)	69.4 (56.0, 78.4)	0.095	69.9 (59.6, 79.7)
Total lymphocytes, N	1.0 (0.7, 1.4)	1.2 (0.9, 1.8)	1.1 (0.8, 1.4)	1.4 (1.0, 1.9)	<0.001	1.1 (0.8, 1.6)
Total lymphocytes, %	17.5 (11.1, 26.9)	22.6 (14.4, 30.5)	21.0 (12.8, 28.3)	20.5 (13.1, 31.2)	0.079	20.5 (12.7, 28.4)
Aspartate amino-transferase (AST), U/L	32.0 (23.5, 42.5)	25.0 (19.0, 41.0)	28.0 (22.0, 42.0)	24.0 (18.0, 38.0)	0.002	27.0 (21.0, 41.0)
Alanine amino-transferase (ALT), U/L	26.0 (18.5, 43.5)	22.0 (14.0, 41.0)	26.5 (16.0, 40.0)	22.0 (14.0, 35.0)	0.072	24.0 (16.0, 40.0)
Bilirubin, mg/L	0.6 (0.5, 0.9)	0.6 (0.5, 0.8)	0.7 (0.5, 1.0)	0.7 (0.5, 1.0)	0.003	0.7 (0.5, 0.9)
Hemoglobin, mg/L	13.8 (12.7, 14.9)	12.9 (11.6, 14.0)	13.7 (12.7, 15.0)	13.2 (11.6, 14.6)	<0.001	13.6 (12.3, 14.7)
Creatinine, mg/L	0.9 (0.8, 1.1)	0.9 (0.7, 1.0)	0.9 (0.8, 1.1)	0.9 (0.7, 1.1)	0.083	0.9 (0.8, 1.1)
D-dimer, mg/L	790.0 (423.0, 1254)	698.0 (436.0, 1245)	660.0 (441.0, 1266)	796.5 (435.0, 1501)	0.923	711.0 (437.0, 1299)
Lactate dehydrogenase, U/L	267.0 (217.0, 368.0)	224.0 (177.0, 282.0)	251.0 (202.0, 326.0)	203.0 (166.0, 264.0)	<0.001	245.0 (192.0, 311.0)
C-reactive protein, mg/L	3.4 (1.5, 9.8)	2.4 (1.2, 7.0)	3.7 (1.6, 8.8)	1.8 (0.2, 4.7)	<0.001	3.0 (1.2, 8.0)
Platelets, 10^9^/L	198.0 (158.5, 274.0)	231.0 (181.0, 309.0)	207.0 (161.0, 276.0)	234.5 (173.5, 289.5)	0.068	217.0 (167.0, 284.5)
Potassium, mmol/L	3.7 (3.4, 3.9)	3.7 (3.3, 3.9)	3.6 (3.4, 3.8)	3.7 (3.4, 4.0)	0.388	3.6 (3.4, 3.9)
Ferritin, mg/L	374.0 (176.0, 839.0)	297.0 (104.0, 637.0)	536.5 (266.5, 1045)	277.5 (128.5, 602.5)	<0.001	427.5 (186.0, 841.0)

All values are expressed as median (IQR).

^a^ Kruskal–Wallis test.

Among the 590 patients included in the analysis, 109 received hydroxychloroquine, 124 received lopinavir/ritonavir, 244 received hydroxychloroquine plus lopinavir/ritonavir, and 113 did not receive any of them (standard of care). The latter group consisted of 44 people who did not start any drug, 55 who started anticoagulants, 25 steroids, 25 azithromycin, and 3 immunomodulatory drugs. The different treatment groups were not homogenous for sex, timing of hospitalization, pneumonia, baseline PaO2/FiO2, and some inflammatory biomarkers. In particular, patients in the SoC group had a higher PaO2/FiO2 at baseline (median 381, IQR 300–467 mmHg) and were less frequently diagnosed with pneumonia (68.1% of them); had lower LDH, AST, CRP, and ferritin; and higher neutrophils and lymphocytes count than the other three groups. Overall, 132 (22%) were treated also with azithromycin, 196 (33%) with corticosteroids at various dosage, 92 (16%) with immunomodulatory drugs, and 277 (47%) received heparin at various dosage. Concomitant use of azithromycin was most prevalent in the lopinavir/r group (*n* = 45, 36 vs. 22% in SoC), while immunomodulatory drugs were most frequently used in the dual antiviral combination (*n* = 51, 21% vs. 3% in SoC) (Table 3).

**TABLE 3:** Endpoint events and other drugs disposition.

	Intervention					
	LPV/r	HCQ	LPV/r + HCQ	SoC	p-value^a^	Total
	*N* = 124	*N* = 109	*N* = 244	*N* = 113		*N* = 590
Events, *n* (%)						
Invasive ventilation	23 (18.5%)	8 (7.3%)	37 (15.2%)	11 (9.7%)	0.04	79 (13.4%)
Death	21 (16.9%)	12 (11.0%)	27 (11.1%)	15 (13.3%)	0.41	75 (12.7%)
Invasive ventilation/death	36 (29.0%)	19 (17.4%)	51 (20.9%)	23 (20.4%)	0.15	129 (21.9%)
Noninvasive ventilation/invasive ventilation/death	38 (30.7%)	23 (21.1%)	61 (25.0%)	26 (23.0%)	0.36	148 (25.0%)
Stop of shedding	50 (47.6%)	41 (47.7%)	117 (56.0%)	42 (59.2%)	0.26	250 (53.1%)
Other drugs, *n* (%)						
Anticoagulants	46 (37.1%)	69 (63.3%)	107 (43.9%)	55 (48.7%)	0.0005	227 (47.0%)
Steroids	64 (51.6%)	26 (23.9%)	81 (33.2%)	25 (22.1%)	<0.0001	196 (33.2%)
Azithromycin	45 (36.3%)	27 (24.8%)	35 (14.3%)	25 (22.1%)	<0.0001	132 (22.4%)
Immunomodulatory drugs	20 (16.1%)	18 (16.5%)	51 (20.9%)	3 (2.7%)	0.0002	92 (15.6%)

^a^ Chi-square test.

### Primary Endpoint: Invasive Ventilation/Death

Overall, 79 patients over 590 (13.4%) underwent invasive ventilation and 75/590 (12.7%) did not survive (Table 3).

**FIGURE 1:**
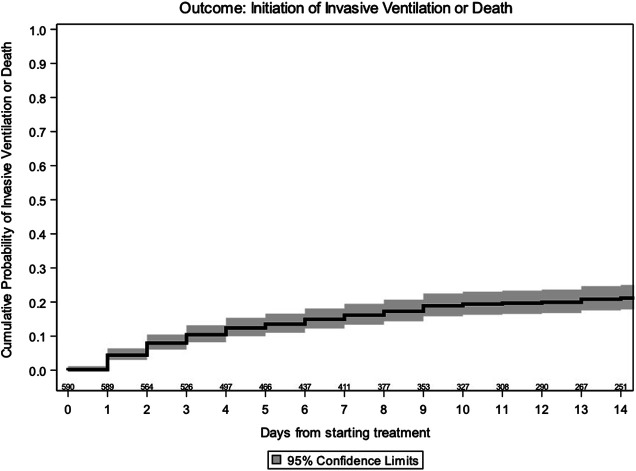
Kaplan–Meier estimate of time to invasive ventilation/death–overall.

**FIGURE 2:**
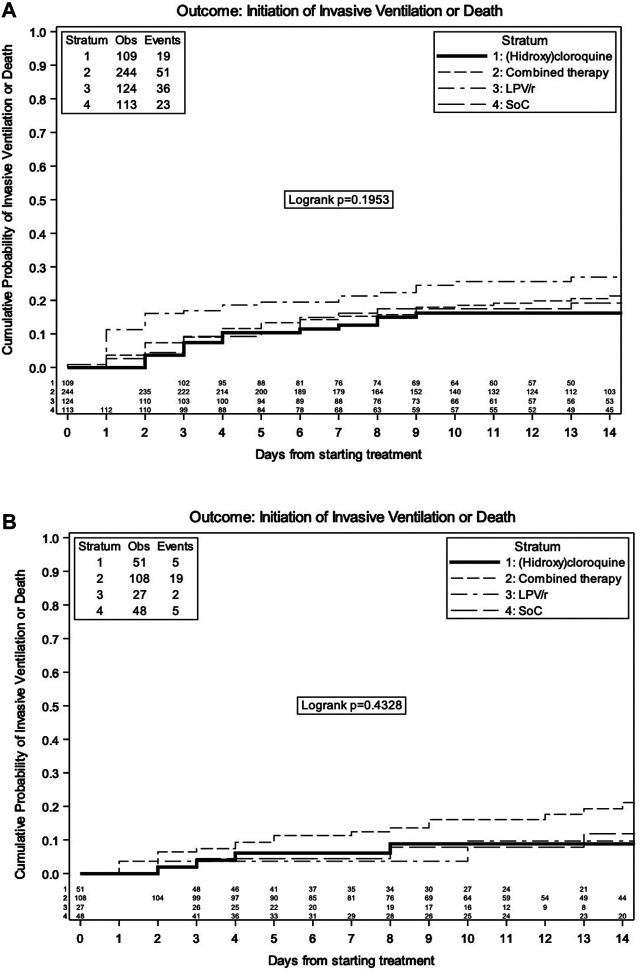
Kaplan–Meier estimate of time to invasive ventilation/death by treatment group in overall population **(A)** and in the strata of moderate patients **(B)**.

By Kaplan–Meier analysis, the estimated probabilities of invasive ventilation or death were 17.3% (95% CI 14.1, 20.4) at 7 days and 21.2% (95% CI 17.6, 24.7) at 14 days in the overall population (Figure 1). The estimated probabilities of invasive ventilation or death at 14 days were 16.2% (95% CI 8.8, 23.5) with SoC, 26.9% (95% CI 18.7, 35.2) with LPV/r, 16.2% (95% CI 8.9, 23.6) with HCQ, and 20.5% (95% CI 15.1, 26.0) with LPV/r + HCQ, without any evidence of a difference between the groups (log rank *p* = 0.20) (Figure 2A).

Even considering only the strata of moderate patients (PaO2/FiO_2_ > 300 mmHg at baseline), no difference among the groups was detected (log rank *p* = 0.43, Figure 2B).

Unadjusted and adjusted marginal relative hazards of invasive ventilation/death from fitting a marginal Cox regression model are shown in Table 4. This model was adjusted for age, gender, presence of comorbidities, duration of symptoms, time-varying use of immunomodulatory drugs, heparin and azithromycin, and censoring using IPW. There was no evidence of a difference in risk of invasive ventilation/death in the three treatment groups when compared to standard of care. The aHR was 1.09 (95% CI 0.60, 1.98) with LPV/r + HCQ, 0.81 (95% CI 0.38, 1.72) with HCQ, and 1.55 (95% CI 0.82, 2.93) with LPV vs. SoC.

**TABLE 4:** HR of invasive ventilation/death from fitting a marginal Cox regression model.

	Unadjusted and adjusted marginal relative hazards of invasive ventilation/death				
	Unadjusted HR (95% CI)	*p*-value	Adjusted^a^ HR (95% CI)	*p*-value	
All patients					
SoC	1.00		1.00		
LPV/r + HCQ	1.01 (0.61, 1.66)	0.972	1.09 (0.60, 1.98)	0.772	
HCQ	0.78 (0.42, 1.44)	0.423	0.81 (0.38, 1.72)	0.584	
LPV/r	1.42 (0.83, 2.45)	0.201	1.55 (0.82, 2.93)	0.173	
Baseline PaO2/FiO2 0–300					
SoC	1.00		1.00		
LPV/r + HCQ	0.51 (0.24, 1.08)		1.41 (0.37, 5.33)		
HCQ	0.53 (0.22, 1.30)		1.48 (0.35, 6.18)		
LPV/r	0.89 (0.41, 1.94)		2.48 (0.65, 9.43)		
				p-value for interaction	
				<0.001	
Baseline PaO2/FiO2 > 300					
SoC	1.00		1.00	
LPV/r + HCQ	1.57 (0.59, 4.17)		1.63 (0.56, 4.78)	
HCQ	0.87 (0.25, 3.00)		0.83 (0.22, 3.18)	
LPV/r	0.67 (0.13, 3.42)		0.73 (0.14, 3.95)	

^a^ Adjusted for age, gender, presence of comorbidities, duration of symptoms and time-varying use of immunomodulatory drugs, azithromycin, steroids, anticoagulants, and censoring using IPW.

NB. The stratified analysis is based on the subset of 396/590 (67%) participants with available PaO2/FiO2 values at baseline.

The risk of invasive ventilation/death in the three groups appeared to vary by PaO2/FiO2, driven by the use of LPV/r or HCQ alone. In fact, for HCQ vs. SoC, the aHR resulted 1.48 (95% CI 0.35, 6.18) in the strata of patients with PaO2/FiO2 < 300 mmHg at baseline and 0.83 (95% CI 0.22, 3.18) in the strata of patients with PaO2/FiO2 > 300 mmHg at baseline, suggesting a more beneficial effect of HCQ in people with less severe disease. Results were similar for LPV/r vs. SoC, with an aHR of 2.48 (95% CI 0.65, 9.43) in the strata of patients with PaO2/FiO2 < 300 mmHg at baseline and 0.73 (95% CI 0.14, 3.95) in the strata of patients with PaO2/FiO2 > 300 mmHg at baseline (p-value for interaction <0.001) (Table 4).

Results were similar in two additional sensitivity analyses. First, after we further controlled for baseline level of inflammation and coagulation in a subset of participants with available values of these markers (ferritin, CRP, and d-dimer), the aHR was 0.84 (95% CI 0.41, 1.70) with LPV/r + HCQ, 0.63 (95% CI 0.26, 1.54) with HCQ, and 1.15 (95% CI 0.54, 2.47) with LPV vs. SoC (Supplementary Table S1). The second sensitivity analysis was done after controlling for pneumonia instead of baseline levels of PaO2/FiO2, to try to remove bias due to imbalance in the severity of disease at entry. The HR in this case were 0.93 (95% CI 0.53, 1.62) with LPV/r + HCQ, 0.60 (95% CI 0.32, 1.12) with HCQ, and 0.65 (95% CI 0.36, 1.20) with LPV vs. SoC.

### Secondary Endpoint i): Viral Shedding

Overall, confirmed negative SARS-CoV-2 PCR in nasal/oropharyngeal swabs, without a relapse, was obtained in 215 patients over 441 during hospitalization (Table 3).

**FIGURE 3:**
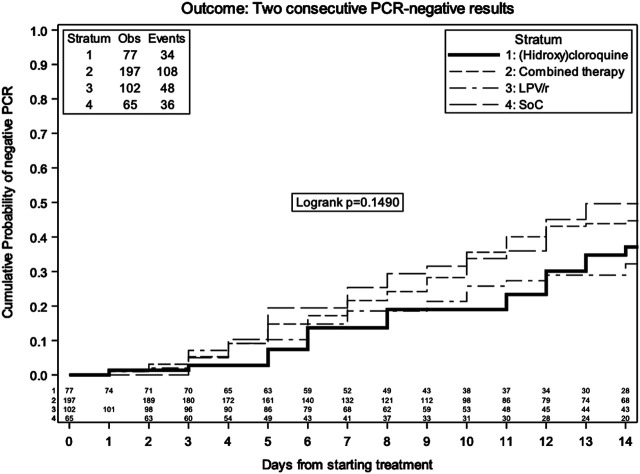
Kaplan–Meier estimate of time to confirmed negative NP/OP swabs by treatment group.

By Kaplan–Meier analysis, the estimated probabilities of confirmed negative SARS-CoV-2 PCR in nasal/oropharyngeal swabs were 22.7% (95% CI 18.5, 26.9) at 7 days and 44.4% (95% CI 38.9, 49.9) at 14 days in the overall population (Figure 3). The estimated probabilities in the different groups at 14 days were 49.7% (95% CI 35.5, 63.8) with SoC, 32.2% (95% CI 21.6, 42.8) with LPV/r, 37.1% (95% CI 23.7, 50.5) with HCQ, and 44.7% (95% CI 36.7, 52.7) with LPV/r, without evidence of a difference between the groups (log rank *p* = 0.15).

Unadjusted and adjusted marginal relative hazards of confirmed negative SARS-CoV-2 PCR in nasal/oropharyngeal swabs from fitting a marginal Cox regression model are shown in Table 5. This model was adjusted for age, gender, presence of comorbidities, duration of symptoms, time-varying use of immunomodulatory drugs, heparin and azithromycin, and censoring using IPW. Again, these data were compatible with the null hypothesis of no difference between the groups. The aHR was 1.09 (95% CI 0.66, 1.79) with LPV/r + HCQ, 0.72 (95% CI 0.41, 1.26) with HCQ, and 0.77 (95% CI 0.44, 1.32) with LPV/r vs. SoC. In the subset of patients with PaO2/FiO2 > 300 mmHg at baseline, the aHR was 0.60 (95% CI 0.32, 1.12) with LPV/r + HCQ, 0.40 (95% CI 0.19, 0.84) with HCQ, and 0.46 (95% CI 0.19, 1.12) with LPV/r vs. SoC.

**TABLE 5:** HR of reversing to PCR negative from fitting a marginal Cox regression model.

	Unadjusted and adjusted marginal relative hazards of reverting from PCR + to PCR negative			
	Unadjusted HR (95% CI)	*p*-value	Adjusted^a^ HR (95% CI)	*p*-value
All patients				
SoC	1.00		1.00	
LPV/r + HCQ	1.11 (0.72, 1.71)	0.625	1.09 (0.66, 1.79)	0.732
HCQ	0.79 (0.48, 1.29)	0.339	0.72 (0.41, 1.26)	0.244
LPV/r	0.82 (0.50, 1.32)	0.409	0.77 (0.44, 1.32)	0.337
Baseline PaO2/FiO2 > 300				
SoC	1.00		1.00	
LPV/r + HCQ	0.62 (0.37, 1.03)		0.60 (0.32, 1.12)	
HCQ	0.42 (0.22, 0.78)		0.40 (0.19, 0.84)	
LPV/r	0.50 (0.21, 1.16)		0.46 (0.19, 1.12)	

^a^ Adjusted for age, gender, presence of comorbidities, duration of symptoms and time-varying use of immunomodulatory drugs, azithromycin, steroids, anticoagulants, and censoring using IPW.

NB. The stratified analysis is based on the subset of 317/471 (67%) participants with available PaO2/FiO2 values at baseline included in the analysis for this endpoint.

Estimates in the PaO2/FiO2 0–300 mmHg stratum could not be calculated due to the small sample size and positivity in the distribution of the weights.

Results were similar when we further adjusted for baseline PaO2/FiO2 levels. The aHR was 1.12 (95% CI 0.68, 1.85) with LPV/r + HCQ, 0.79 (95% CI 0.45, 1.36) with HCQ, and 0.78 (95% CI 0.45, 1.36) with LPV/r vs. SoC, and these risks did not vary by stratification for duration of symptoms (more or less than 9 days from symptoms’ onset) (Supplementary Table S2).

### Secondary Endpoint ii): Noninvasive or Invasive Ventilation or Death

Overall, 101 patients over 590 (17.1%) underwent noninvasive or invasive ventilation (Table 3).

**FIGURE 4:**
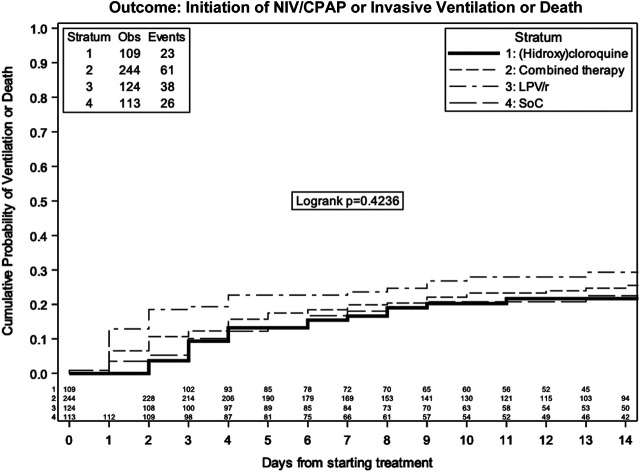
Kaplan–Meier estimate of time to ventilation/death by treatment group.

By Kaplan–Meier analysis, the estimated probabilities of noninvasive/invasive ventilation or death was 20.8% (95% CI 17.4, 24.2) at 7 days and 25.7% (95% CI 21.8, 29.6) at 14 days in the overall population (Figure 4). The estimated probabilities of noninvasive or invasive ventilation or death at 14 days were 18.0% (95% CI 10.4, 25.7) with SoC, 24.7% (95% CI 18.9, 30.5) with LPV/r + HCQ, 20.3% (95% CI 12.2, 28.3) with HCQ, and 29.3% (95% CI 20.8−, 7.8) with LPV/r, without evidence of a difference between SOC, LPV/r, HCQ, and LPV/r + HCQ (log rank p = 0.42).

## Discussion

Herein, we report a retrospective study with real-world data collected from routine care to assess the clinical and virological efficacy of hydroxychloroquine, lopinavir/ritonavir, or the combination of hydroxychloroquine plus lopinavir/ritonavir vs. SoC in a population of 590 patients admitted to our hospital for COVID-19 infection. We found that none of the antivirals investigated or their combination were associated with a reduction of invasive ventilation or death 14 days after starting of therapy compared with standard of care alone. Additionally, a reduction in estimated probability of any ventilation (noninvasive or invasive ventilation) or death was not demonstrated. Even in terms of ending of the viral shedding, in the subgroup of patients with positivity to SARS-CoV-2 in nasal/oropharyngeal swabs, we found no benefit with hydroxychloroquine, lopinavir/ritonavir, or the combination of hydroxychloroquine plus lopinavir/ritonavir in comparison to the standard of care.

Our population is well-characterized and clinical features of our patients were consistent with many other reports, with a predominance of men, mean aged in the seventh decade of life. In contrast with other reports, we found a lower prevalence of comorbidities in our population (Docherty et al., 2020; Richardson et al., 2020), but higher number of symptomatic patients with fever (Richardson et al., 2020).

As expected, results of the comparison between HCQ or LPV/r monotherapy and SoC were similar to those of randomized trials, where no advantage in the use of HCQ or LPV/r was observed (Cavalcanti et al., 2020; NIH, 2020b; Recovery Randomised Evaluation of COVID-19 Therapy 2020a; Recovery Randomised Evaluation of COVID-19 Therapy, 2020b; World Health Organization, 2020). All our analyses took into account heparin use and corticosteroids, and this is particularly relevant in light of the possible association between anticoagulant treatment and decreased mortality in severe COVID-19 and above all in light of recently published data from randomized and observational studies about benefits of corticosteroids in terms of clinical evolution (Group TRC, 2020; Salton et al., 2020) and mortality (Fadel et al., 2020; Fernández Cruz et al., 2020; Group TRC, 2020; Salton et al., 2020; Wu et al., 2020).

The risk of invasive ventilation/death in the groups appeared to vary by PaO2/FiO2 at baseline; its putative mechanism was not clear and could deserve further investigation. Antivirals were administered approximately 9 days after symptoms’ initiation, similarly to other studies (Cao et al., 2020; Goldman et al., 2020). It is possible that therapies that may limit viral replication may be more effective earlier in the course of the disease, so this could explain our signal for a greater potential benefit of HCQ and LPV/r vs. SoC in the subset of people with less severe disease. According to Hung et al. (Hung et al., 2020), antivirals could also potentially have a role in reducing viral shedding, but we found no such evidence in our cohort. The analysis has been prompted by a recent RCT showing the beneficial effect of combining antivirals for the treatment of COVID-19 patients (Hung et al., 2020). Indeed, these strategies have been seldom compared in randomized studies and more research is needed. Of note, the effectiveness of the triple combination in that study was mainly ascribed to the use of interferon beta, which has potential to prevent SARS-CoV-2 from shutting down the host innate immunity in the first few days from infection (Richardson et al., 2020). Interferon beta was not used in this case, so this might explain why we could not replicate these earlier results. In general, COVID-19 is a complex disease from the standpoint of pathogenesis with different stages, so broad comparisons of drug A vs. B might not be as useful as trials designed to compare interventions tailored to patients following specific pathogenic pathways (e.g., cytokines storm as opposed to microcirculatory platelet aggregation, etc.). Indeed, the case-mix of our study population was quite heterogeneous. The ending of viral shedding seemed to be possibly favored by HCQ, but only in patients with moderate COVID-19 and not confirmed in a further analysis adjusted for PaO2/FiO2 at baseline and stratified for duration of symptoms. Anyway, this is a subset analysis, so p-values should be considered with caution. Even previously published data showed conflicting results, but considering all the evidence cumulated to date on the lack of efficacy of HCQ, its beneficial role on SARS-COV-2 shedding should be considered unlikely (NIH, 2020a; Tang et al., 2020; Huang et al., 2020).

Our study presents some limitations. First of all because it is retrospective and observational, we cannot rule out unmeasured confounding. The analysis also relies on specific assumptions regarding the underlying causal structure of the data (time-fixed and time-varying confounding factors) and the linear predictor of the model, with or without interaction terms, to be correctly specified. Thus, it is possible that a key variable was missing in our propensity scores; however, after further controlling for markers of inflammation/coagulation, results were similar. Also, although there was a standard treatment protocol in place, untreated patients in the SoC group might have been a selected population in which treatment was withheld because of predicted poor prognosis or conversely because of initial better evaluation. In fact, the SoC group showed healthier profiles at baseline, in terms of inflammation biomarkers and of leukocyte count and a rate of admission to the ICU which was comparable with the overall mortality (10.3% vs. 9.7%), suggesting that all participants had been equally considered for critical care. Further, other sub-analyses have been performed, taking into account a diagnosis of pneumonia and the baseline difference in biomarkers among groups and similar results were obtained. Indeed, in our population, patients allocated to SoC showed a much less COVID-19 disease severity and our propensity score adjustment should have minimized this imbalance. Also, because time zero of the survival analyses was the date of starting treatment, immortal bias also cannot be completely ruled out. However, the average time from hospital admission to therapy initiation was <1 day for 75% of the study population. Finally, safety data (i.e., risk of arrhythmia in people receiving HCQ) have not been analyzed in this work.

On the other hand, key strengths of this work were the detailed characterization of the study population, including the coadministered drugs, the possibility of comparing combination treatment strategies seldom investigated in randomized studies, and the use of a sophisticated counterfactual prediction framework to appropriately control for time-fixed and time-vary confounding factors.

## Conclusion

In our retrospective analysis of real-life data of hospitalized patients with mild-to-severe COVID-19, we did not find a significant difference in clinical and virological outcome among lopinavir/ritonavir, hydroxychloroquine, and lopinavir/ritonavir plus hydroxychloroquine or standard of care. Our results are consistent with those of randomized trials comparing mono-antiviral treatment arms vs. placebo which led to the recommendation against the use of these antivirals for treatment of COVID-19 patients in national and international guidelines. Indeed, some of the early RCTs were of poor quality and risk of bias was high, but larger more recent and reliable studies confirmed these results. Additional RCTs specifically addressing the timing of initiation of these and other interventions according to patients’ disease course and specific pathogenic pathways as well as the use a combination of approaches are further needed.

## Collaborative Authors

Collaborators Members of the National Institute for Infectious Diseases (INMI) ReCOVeRI study group: Maria Alessandra Abbonizio, Amina Abdeddaim, Elisabetta Agostini, Chiara Agrati, Fabrizio Albarello, Gioia Amadei, Alessandra Amendola, Andrea Antinori, Maria Assunta Antonica, Mario Antonini, Tommaso Ascoli Bartoli, Francesco Baldini, Raffaella Barbaro, Barbara Bartolini, Rita Bellagamba, Martina Benigni, Nazario Bevilacqua, Gianluigi Biava, Michele Bibas, Licia Bordi, Veronica Bordoni, Evangelo Boumis, Marta Branca, Rosanna Buonomo, Donatella Busso, Marta Camici, Paolo Campioni, Flaminia Canichella, Maria Rosaria Capobianchi, Alessandro Capone, Cinzia Caporale, Emanuela Caraffa, Ilaria Caravella, Fabrizio Carletti, Concetta Castilletti, Adriana Cataldo, Stefano Cerilli, Carlotta Cerva, Roberta Chiappini, Pierangelo Chinello, Maria Assunta Cianfarani, Carmine Ciaralli, Claudia Cimaglia, Nicola Cinicola, Veronica Ciotti, Stefania Cicalini, Francesca Colavita, Angela Corpolongo, Massimo Cristofaro, Salvatore Curiale, Alessandra D’Abramo, Cristina Dantimi, Alessia De Angelis, Giada De Angelis, Maria Grazia De Palo, Federico De Zottis, Virginia Di Bari, Rachele Di Lorenzo, Federica Di Stefano, Gianpiero D’Offizi, Davide Donno, Francesca Evangelista, Francesca Faraglia, Anna Farina, Federica Ferraro, Lorena Fiorentini, Andrea Frustaci, Matteo Fusetti, Vincenzo Galati, Roberta Gagliardini, Paola Gallì, Gabriele Garotto, Ilaria Gaviano, Saba Gebremeskel Tekle, Maria Letizia Giancola, Filippo Giansante, Emanuela Giombini, Guido Granata, Maria Cristina Greci, Elisabetta Grilli, Susanna Grisetti, Gina Gualano, Fabio Iacomi, Marta Iaconi, Giuseppina Iannicelli, Carlo Inversi, Giuseppe Ippolito, Eleonora Lalle, Maria Elena Lamanna, Simone Lanini, Daniele Lapa, Luciana Lepore, Raffaella Libertone, Raffaella Lionetti, Giuseppina Liuzzi, Laura Loiacono, Andrea Lucia, Franco Lufrani, Manuela Macchione, Gaetano Maffongelli, Alessandra Marani, Luisa Marchioni, Andrea Mariano, Maria Cristina Marini, Micaela Maritti, Annelisa Mastrobattista, Ilaria Mastrorosa, Giulia Matusali, Valentina Mazzotta, Paola Mencarini, Silvia Meschi, Francesco Messina, Sibiana Micarelli, Giulia Mogavero, Annalisa Mondi, Marzia Montalbano, Chiara Montaldo, Silvia Mosti, Silvia Murachelli, Maria Musso, Michela Nardi, Assunta Navarra, Emanuele Nicastri, Martina Nocioni, Pasquale Noto, Roberto Noto, Alessandra Oliva, Ilaria Onnis, Sandrine Ottou, Claudia Palazzolo, Emanuele Pallini, Fabrizio Palmieri, Giulio Palombi, Carlo Pareo, Virgilio Passeri, Federico Pelliccioni, Giovanna Penna, Antonella Petrecchia, Ada Petrone, Nicola Petrosillo, Elisa Pianura, Carmela Pinnetti, Maria Pisciotta, Pierluca Piselli, Silvia Pittalis, Agostina Pontarelli, Costanza Proietti, Vincenzo Puro, Paolo Migliorisi Ramazzini, Alessia Rianda, Gabriele Rinonapoli, Silvia Rosati, Dorotea Rubino, Martina Rueca, Alberto Ruggeri, Alessandra Sacchi, Alessandro Sampaolesi, Francesco Sanasi, Carmen Santagata, Alessandra Scarabello, Silvana Scarcia, Vincenzo Schininà, Paola Scognamiglio, Laura Scorzolini, Giulia Stazi, Giacomo Strano, Fabrizio Taglietti, Chiara Taibi, Giorgia Taloni, Tetaj Nardi, Roberto Tonnarini, Simone Topino, Martina Tozzi, Francesco Vaia, Francesco Vairo, Maria Beatrice Valli, Alessandra Vergori, Laura Vincenzi, Ubaldo Visco-Comandini, Serena Vita, Pietro Vittozzi, Mauro Zaccarelli, Antonella Zanetti and Sara Zito.

## Data Availability

The raw data supporting the conclusions of this article will be made available by the authors, without undue reservation.

## References

[B1] Bobrowski T. Chen L. Eastman R. T. Itkin Z. Shinn P. Chen C. Z. (2021). Synergistic and antagonistic drug combinations against SARS-CoV-2. Mol. Ther. 29 (2), 873–885. 10.1016/j.ymthe.2020.12.016 33333292 PMC7834738

[B2] Cao B. Wang Y. Wen D. Liu W. Wang J. Fan G. (2020). A trial of lopinavir–ritonavir in adults hospitalized with severe covid-19. N. Engl. J. Med. 382, 1787–1799. 10.1056/NEJMoa200128 32187464 PMC7121492

[B3] Cavalcanti A. B. Zampieri F. G. Rosa R. G. Azevedo L. C. P. Veiga V. C. Avezum A. (2020). Hydroxychloroquine with or without azithromycin in mild-to-moderate covid-19. N. Engl. J. Med. 383, 2041–2052. 10.1056/NEJMoa2019014 32706953 PMC7397242

[B4] Chowdhury M. S. Rathod J. Gernsheimer J. (2020). A rapid systematic review of clinical trials utilizing chloroquine and hydroxychloroquine as a treatment for COVID-19. Acad. Emerg. Med. 27 (6), 493–504. 10.1111/acem.14005 32359203 PMC7267507

[B5] European Centre for Disease Prevention and Control (2020). Outbreak of novel coronavirus disease 2019 (COVID-19): increased transmission globally–fifth update. Stockholm, Sweden: ECDC (Accessed March 2, 2020).

[B6] Docherty A. B. Harrison E. M. Green C. A. Hardwick H. E. Pius R. Norman L. (2020). Features of 20 133 UK patients in hospital with covid-19 using the ISARIC WHO Clinical Characterisation Protocol: prospective observational cohort study. BMJ 369, 1–12. 10.1136/bmj.m1985 PMC7243036 32444460

[B7] Fadel R. Morrison A. R. Vahia A. Smith Z. R. Chaudhry Z. Bhargava P. (2020). HFC-19 MTF. Early short course corticosteroids in hospitalized patients with COVID-19. Clin. Infect. Dis. 71 (16), 2114–2120. 10.1093/aobpla/ply036/5033327 32427279 PMC7314133

[B8] Fernández Cruz A. Ruiz-Antorán B. Muñoz Gómez A. Sancho López A. Mills Sánchez P. Centeno Soto G. A. (2020). Impact of glucocorticoid treatment in sars-cov-2 infection mortality: a retrospective controlled cohort study. Antimicrob. Agents Chemother. 64, e01168-20. 10.1101/2020.05.22.20110544 PMC7449182 32571831

[B9] Ford N. Vitoria M. Rangaraj A. Norris S. L. Calmy A. Doherty M. (2020). Systematic review of the efficacy and safety of antiretroviral drugs against SARS, MERS, or COVID-19: initial assessment. J. Int. AIDS Soc. 23, e25489. 10.1002/jia2.25489 32293807 PMC7158851

[B10] Goldman J. D. Lye D. C. B. Hui D. S. Marks K. M. Bruno R. Montejano R. (2020). Remdesivir for 5 or 10 Days in patients with severe covid-19. N. Engl. J. Med. 383 (19), 1827–1837. 10.1056/NEJMoa2015301 32459919 PMC7377062

[B11] Group TRC (2020). Dexamethasone in hospitalized patients with covid-19 — preliminary report. N. Engl. J. Med 384, 693–704. 10.1056/NEJMoa2021436 32678530 PMC7383595

[B12] Huang M. Li M. Xiao F. Pang P. Liang J. Tang T. (2020). Preliminary evidence from a multicenter prospective observational study of the safety and efficacy of chloroquine for the treatment of COVID-19. Natl. Sci. Rev. 7, 1428–1436. 10.1101/2020.04.26.20081059 PMC7313782 34676087

[B13] Hung I. F. Lung K. Tso E. Y. Liu R. Chung T. W. Chu M. (2020). Triple combination of interferon beta-1b, lopinavir – ritonavir, and ribavirin in the treatment of patients admitted to hospital with COVID-19 : an open-label, randomised, phase 2 trial. Lancet 395, 1695–1704. 10.1016/S0140-6736(20)31042-4 32401715 PMC7211500

[B14] Li Y. Xie Z. Lin W. Cai W. Wen C. Guan Y. (2020). Efficacy and safety of lopinavir/ritonavir or arbidol in adult patients with mild/moderate COVID-19: an exploratory randomized controlled trial. Med (N Y) 1 (1), 105–113.e4. 10.1016/j.medj.2020.04.001 32838353 PMC7235585

[B15] NIH (2020a). Coronavirus Disease 2019 (COVID-19) treatment guidelines. Available at: https://covid19treatmentguidelines.nih.gov/ (Accessed December 7, 2020), 130. 34003615

[B16] NIH (2020b) NIH halts clinical trial of hydroxychloroquine. Available at: www.nih.gov/news-events/news-releases/nih-halts-clinical-trial-hydroxychloroquine/ (Accessed June 20, 2020).

[B17] Recovery Randomised Evaluation of COVID-19 Therapy (2020a). No clinical benefit from use of hydroxychloroquine in hospitalised patients with COVID-19. Available at: www.recoverytrial.net/news/statement-from-the-chief-investigators-of-the-randomised-evaluation-of-covid-19-therapy-recovery-trial-on-hydroxychloroquine-5-june-2020-no-clinical-benefit-from-use-of-hydroxychloroquine-in-hospitalised-patients-with-covid-19 n.d. (Accessed June 5, 2020).

[B18] Recovery Randomised Evaluation of COVID-19 Therapy (2020b). No clinical benefit from use of lopinavir-ritonavir in hospitalised COVID-19 patients studied in recovery. Available at: www.recoverytrial.net/news/no-clinical-benefit-from-use-oflopinavir-ritonavir-in-hospitalised-covid-19-patients-studied-in-recovery on 01/07/2020 (Accessed July 01, 2020).

[B19] Richardson S. Hirsch J. S. Narasimhan M. Crawford J. M. McGinn T. Davidson K. W. (2020). Presenting characteristics, comorbidities, and outcomes among 5700 patients hospitalized with COVID-19 in the New York city area. JAMA 323, 2052–2059. 10.1001/jama.2020.6775 32320003 PMC7177629

[B20] Salton F. Confalonieri P. Santus P. Harari S. Scala R. Lanini S. (2020). Prolonged low-dose methylprednisolone in patients with severe COVID-19 pneumonia. MedRxiv 7, 2020. 10.1101/2020.06.17.20134031 PMC7543560 33072814

[B21] Tang W. Cao Z. Han M. Wang Z. Chen J. Sun W. (2020). Hydroxychloroquine in patients with mainly mild to moderate coronavirus disease (2019) : open label, randomised controlled trial. BMJ 369, m1849. 10.1136/bmj.m1849 32409561 PMC7221473

[B22] Tobaiqy M. Qashqary M. Al-Dahery S. Mujallad A. Hershan A. A. Kamal M. A. (2020). Therapeutic management of patients with COVID-19: a systematic review. Infect. Prev. Pract. 2, 100061. 10.1016/j.infpip.2020.100061 PMC7162768 34316558

[B23] Vincent M. J. Bergeron E. Benjannet S. Erickson B. R. Rollin P. E. Ksiazek T. G. (2005). Chloroquine is a potent inhibitor of SARS coronavirus infection and spread. Virol. J. 2, 1–10. 10.1186/1743-422x-2-69 16115318 PMC1232869

[B24] WHO Solidarity Trial Consortium Pan H. Peto R. Henao-Restrepo A. M. Preziosi M. P. Sathiyamoorthy V. (2020). Repurposed antiviral drugs for covid-19-interim WHO solidarity trial results. N. Engl. J. Med. 384, 497–511. 10.1056/NEJMoa2023184 33264556 PMC7727327

[B25] World Health Organization (2020). WHO discontinues hydroxychloroquine and lopinavir/ritonavir treatment arms for COVID-19. Available at: https://www.who.int/news-room/detail/04-07-2020-who-discontinues-hydroxychloroquine-and-lopinavir-ritonavir-treatment-arms-for-covid-19/ (Accessed July 04, 2020)

[B26] Wu C. Chen X. Cai Y. Xia J. A. Zhou X. Xu S. (2020). Risk factors associated with acute respiratory distress syndrome and death in patients with coronavirus disease 2019 pneumonia in Wuhan, China. JAMA Intern. Med. 180, 934–943. 10.1001/jamainternmed.2020.0994 32167524 PMC7070509

