# Supplementary material for: No Efficacy of the Combination of Lopinavir/Ritonavir Plus Hydroxychloroquine Versus Standard of Care in Patients Hospitalized With COVID-19: A Non-Randomized Comparison
Source: Front Pharmacol. 2021 Apr 22;12:621676. doi: 10.3389/fphar.2021.621676 (PMC8100580; doi:10.3389/fphar.2021.621676)
Supplement: Supplementary file 1 [file datasheet1.docx]

**Supplementary Table 1: HR of invasive ventilation/death from fitting a marginal Cox regression model**

|  | Unadjusted and adjusted marginal relative hazards of invasive ventilation/death | | | |
| --- | --- | --- | --- | --- |
|  | **Unadjusted HR (95% CI)** | **p-value** | **Adjusted^*^ HR (95% CI)** | **p-value** |
|  | **All patients** | | | |
| SoC | 1.00 |  | 1.00 |  |
| LPV/r+HCQ | 1.01 (0.61, 1.66) | 0.972 | 0.84 (0.41, 1.70) | 0.620 |
| HCQ | 0.78 (0.42, 1.44) | 0.423 | 0.63 (0.26, 1.54) | 0.314 |
| LPV/r | 1.42 (0.83, 2.45) | 0.201 | 1.15 (0.54, 2.47) | 0.718 |
|  | | | | |

^*^adjusted for age, gender, presence of comorbidities, duration of symptoms, pneumonia at baseline, baseline CRP, ferritin and d-dimer and time-varying use of immuno-modulatory drugs, azithromycin, steroids, anticoagulants and censoring using IPW.

Supplementary Table 2: HR of reversing to PCR negative from fitting a marginal Cox regression model - SoC as comparator stratified by duration of symptoms

|  | Unadjusted and adjusted marginal relative hazards of reverting to PCR-negative | | | |
| --- | --- | --- | --- | --- |
|  | **Unadjusted HR (95% CI)** | **p-value** | **Adjusted^*^ HR (95% CI)** | **p-value** |
|  | **All patients** | | | |
| SoC | 1.00 |  | 1.00 |  |
| LPV/r+HCQ | 1.11 (0.72, 1.71) | 0.625 | 1.12 (0.68, 1.85) | 0.655 |
| HCQ | 0.79 (0.48, 1.29) | 0.339 | 0.79 (0.45, 1.36) | 0.391 |
| LPV/r | 0.82 (0.50, 1.32) | 0.409 | 0.78 (0.45, 1.36) | 0.386 |
|  | **Duration of symptoms 0-9 days** | | | |
| SoC | 1.00 |  | 1.00 |  |
| LPV/r+HCQ | 1.63 (0.73, 3.60) |  | 1.33 (0.62, 2.86) |  |
| HCQ | 0.91 (0.40, 2.10) |  | 0.76 (0.34, 1.71) |  |
| LPV/r | 1.01 (0.44, 2.30) |  | 0.84 (0.38, 1.87) |  |
|  |  |  |  | **p-value for interaction** |
|  |  |  |  | 0.900 |
|  | **Duration of symptoms >9 days** | | | |
| SoC | 1.00 |  | 1.00 |  |
| LPV/r+HCQ | 0.73 (0.40, 1.33) |  | 1.59 (0.49, 5.17) |  |
| HCQ | 0.52 (0.21, 1.29) |  | 1.16 (0.30, 4.49) |  |
| LPV/r | 0.64 (0.31, 1.32) |  | 1.41 (0.41, 4.83) |  |

| ^*^adjusted for age, gender, baseline PaO2/FiO2, presence of comorbidities and time-varying use of immuno-modulatory drugs, azithromycin, steroids, anticoagulants and censoring using IPW |
| --- |
